# Monitoring the occurrence of pests on cover crops

**DOI:** 10.1016/j.dib.2026.112690

**Published:** 2026-03-16

**Authors:** Julie Sobotková, Pavel Kolařík, Antonín Kintl, Ondřej Malíček, Oldřich Látal, Igor Huňady, Zuzana Kubíková, Martina Dočkalíková, Jakub Elbl

**Affiliations:** a Agricultural Research, Ltd., Zahradní 1, 664 41 Troubsko, Czech Republic; b Department of Agrochemistry, Soil Science, Microbiology and Plant Nutrition, Faculty of AgriSciences, Mendel University in Brno, Zemědělská 1, Brno 61300, Czech Republic; c Department of Agrosystems and Bioclimatology, Faculty of AgriSciences, Mendel University in Brno, Zemědělská 1, 613 00 Brno, Czech Republic

**Keywords:** Cover crops, Insect pests, BBCH stages, Damage to leaf area, Cabbage-stem flea beetle, Flea beetle Phyllotreta sp., Weevil Sitona sp., Bird cherry-oat aphid, Turnip sawfly larvae

## Abstract

The Dataset brings results of a 3-year field evaluation (2022–2024) of the occurrence of insect pests and damage caused by them to ten cover crop species – Watercress (*Lepidium sativum* L.), Oil radish (*Raphanus sativus* L.), Mustard (*Sinapis alba* L.), Purple tansy (*Phacelia tanacetifolia* B.), Crimson clover (*Trifolium incarnatum* L.), Millet (*Panicum miliaceum* L.), Buckwheat (*Fagopyrum esculentum* L.), Fenugreek (*Trigonella foenum - graecum* L.), Hungarian vetch (*Vicia pannonica* Crantz.), and Safflower (*Carthamus tinctorius* L.), grown after the harvest of winter wheat. Observations made in the respective years revealed six key pest species whose abundance was determined or leaf area damage converted to one plant. In order to allow an interspecific comparison when applying two different evaluation methods, all variables were standardized using the min-max (0–1) method and a Composite Susceptibility Index (CSI) was calculated. The data set contains the mean CSI value including zeros – no occurrence (overall risk of infestation and damage) as well as without zeros (intensity of infestation and damage), 95 % confidence intervals and categorial susceptibility classes. Resulting data provide a quantitative basis for the identification of cover crops less attractive to insect pests and for the evaluation of ecological stability of the stands of cover crops in conditions of Central Europe.

Specifications Table

**Table:** 

Subject	Agronomy and Crop Science
Specific subject area	Insect pests on cover crops and assessment of the extent of damage to plants caused by the pests.
Type of data	Raw data, Tables, Figures
Data collection	Visual monitoring and inspection of experimental stands including the identification of key insect pest species, their abundance and assessment of damage caused to plants by their feeding in some phenological stages.
Data source location	Research Institute for Fodder Crops, Ltd. Troubsko 664 41 Troubsko Czech Republic 49.1730775 N, 16.5057914E
Data accessibility	Repository name: Mendeley Data identification number: 10.17632/trf82cn32c.1 Direct URL to data: https://data.mendeley.com/datasets/trf82cn32c/1
Related research article	The presented paper contains data that have not been published anywhere as a part of another research and represent a separate complete set of data.

## 1 Value of the Data

• Some insect pests on cover crops should be considered important vectors of diseases and they should also apply to pests on the main field crops. If the infestation of cover crops is underestimated, there is a risk of the massive overpopulation of pests during hibernation and a danger to key winter cereals or rape after cover crop harvest. Subsequently, conditions suitable for their development and harmfulness increase in the rural landscape with the increasing areas and abundance of cover crops (so-called Green bridges).
• The presented set of data is unique as it brings information about susceptibility to the effect of so-called green bridge. This effect consists in the creation of environment in cover crops that is suitable for the growth and reproduction of pests and their dislocation to the stand of main crop cultivated after the cover crop. The data set can be used also by other researchers to study these cover crop species that are grown worldwide.
• The data set includes detail records on the occurrence of pests as well as the follow-up evaluation of damage to plants in ten commonly grown cover crops capturing the key interactions between insects and host plants in agrosystems of Central Europe. These detailed data make it possible to compare the individual species and may bring new knowledge about agro-ecological relations on the level of species, genera and families.
• Standardized methods of evaluation include both the determination of pests and the quantitative damage assessment based on EPPO PP methodologies 1/218(2), 1/20(3), 1/291(1), 1/73(4), 1/233(1) and 1/60(3) with a modification made in the form of assessments on individual dates in relation to the plant development stage. This makes it possible for other researchers to replicate these protocols, to extend them or to include them into meta-analyses of similar field experiments. The well-structured data can also be integrated with wider data bases and prediction models on the occurrence of pests.
• The presented data on pests and cover crops open up possibilities for reviewing the degree of pest pressure and for the calibration of population models. Researchers can use the data set in simulations, in drafting recommendations for specific regions or for the development of new technologies such as AI tools for recognizing pests in other crops.
• Results can be also used as a complex set of data on the occurrence of specific insect species in their collection for a follow-up research, e.g. laboratory breeding for experiments in controlled conditions or for the tests of sensitivity to active substances of plant protection preparations according to IRAC methodologies.
• The data set is useful for experts from the field of entomology, plant protection, agronomy and climatology and can be used for the comparison between studies, improvement of prediction algorithms or development of intelligent pest monitoring systems, as shown by the current research in the field of smart agriculture.
• By including data on the occurrence of pests and evaluation of damages caused by them in various cover crops, the data set is a valuable source for long-term studies, applications in evolutionary ecology and assessment of pests adaptation in changing climatic or agro-ecological conditions.

## 2 Background

The preparation of this data set was motivated by the need to monitor the occurrence of insect species (pests) as well as by the related detail quantification of damage to the plants of ten cover crops species at certain stages of development in central European agro-ecosystems. Theoretical knowledge about the significance of cover crops preceding this research was focused on increasing biodiversity, soil protection and support to sustainable production. Methodologically, the data was obtained by repeated visual inspections of stands, which included identification of pest species, their abundance and assessment of damage caused by their feeding on plants at certain phenological stages. This approach was based on proven methods of monitoring pests in the field crops and it was combined with standardized protocols ensuring repeatability and comparability of results. Thus the Dataset brings a detailed view of interactions between pests and cover crops in the specific environmental and agrotechnical context.

As a result of the evaluation of pest occurrence and damage to plants, modifications can be proposed in the composition of cover crops, their sowing dates, mechanical soil treatment or termination of their vegetation. In a majority of cases, an appropriate selection of species can minimize the occurrence and harmfulness of insect pests so that basic functions of cover crops in the rotation are not threatened.

As to the connection with the original research papers, these data extend and complement the published research findings with quantitative information that can be used in validation, comparison or further analyses related to the development of pest populations and strategy of their control.

## 3 Data Description

**Fig. 1:**
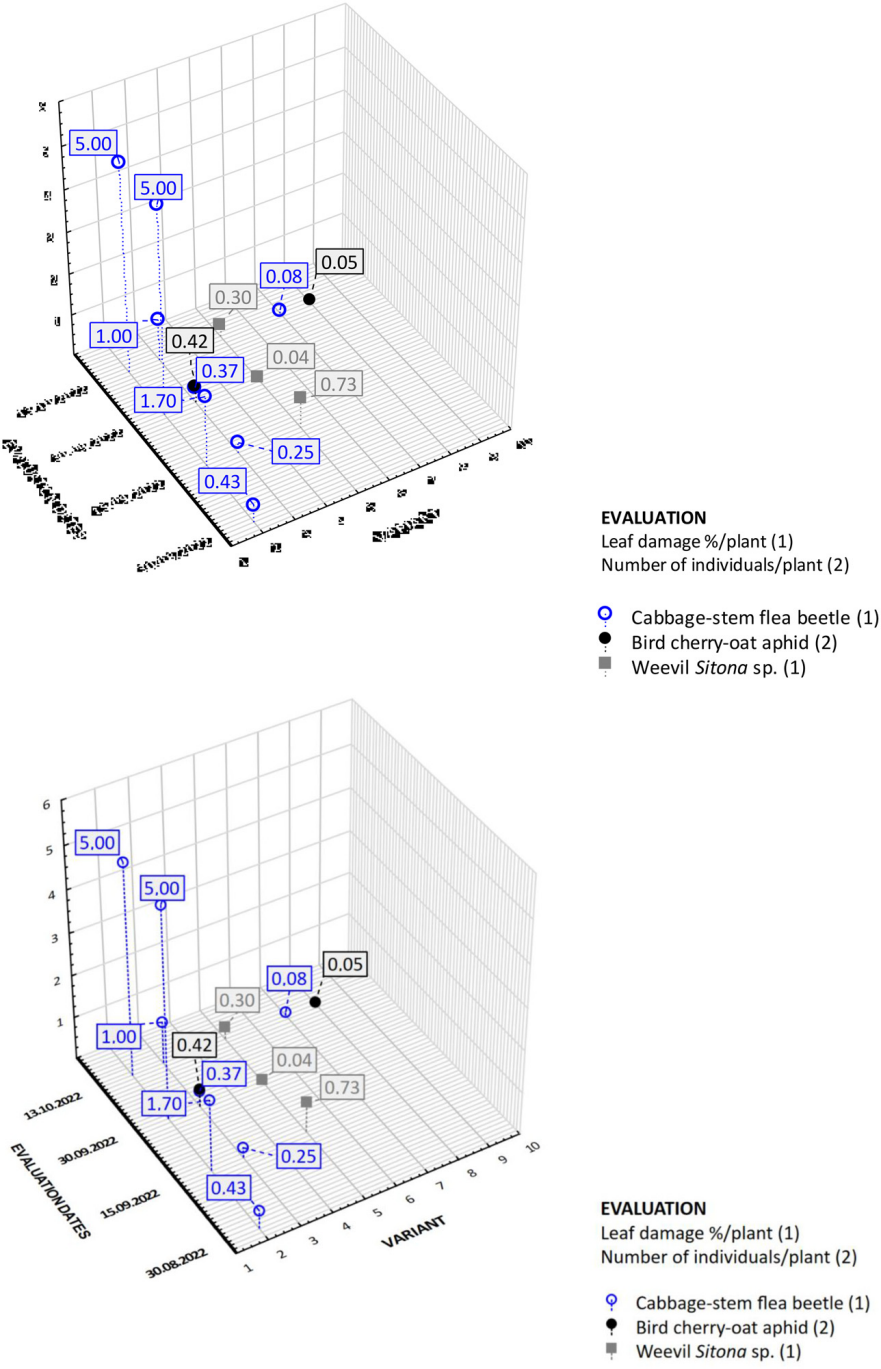
3D Scatterplot of multiple variables against evaluation dates and variants (cover crops) in 2022. Variants: 1 Watercress (*Lepidium sativum* L.), 2 Oil radish (*Raphanus sativus* L.), 3 Mustard (*Sinapis alba* L.), 4 Purple tansy (*Phacelia tanacetifolia* B.), 5 Crimson clover (*Trifolium incarnatum* L.), 6 Millet (*Panicum miliaceum* L.), 7 Buckwheat (*Fagopyrum esculentum* L.), 8 Fenugreek (*Trigonella foenum - graecum* L.), 9 Hungarian vetch (*Vicia pannonica Crantz*.), 10 Safflower (*Carthamus tinctorius* L.).

**Fig. 2:**
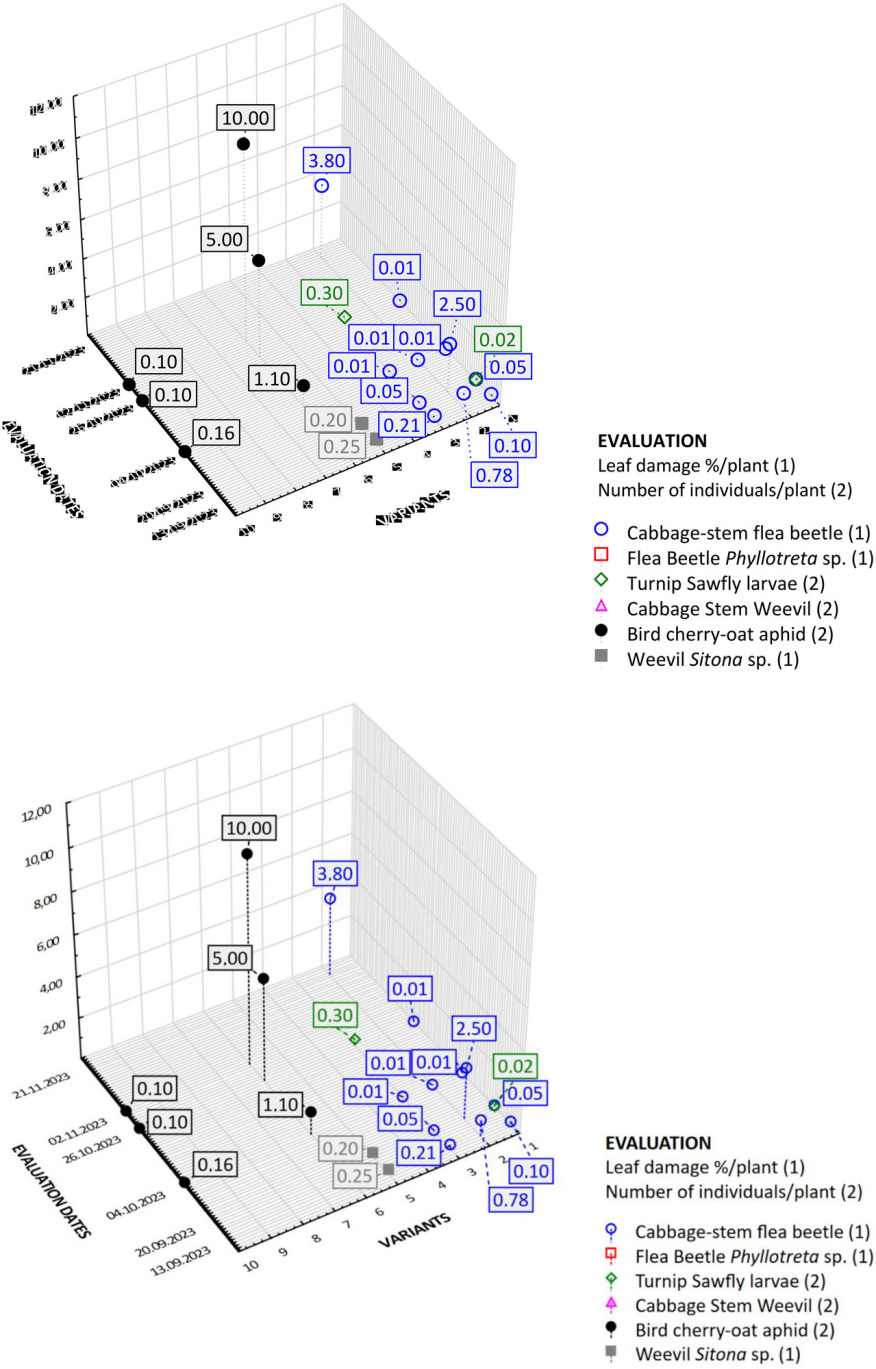
3D Scatterplot of multiple variables against evaluation dates and variants (cover crops) in 2023. Variants: 1 Watercress (*Lepidium sativum* L.), 2 Oil radish (*Raphanus sativus* L.), 3 Mustard (*Sinapis alba* L.), 4 Purple tansy (*Phacelia tanacetifolia* B.), 5 Crimson clover (*Trifolium incarnatum* L.), 6 Millet (*Panicum miliaceum* L.), 7 Buckwheat (*Fagopyrum esculentum* L.), 8 Fenugreek (*Trigonella foenum - graecum* L.), 9 Hungarian vetch (*Vicia pannonica Crantz*.), 10 Safflower (*Carthamus tinctorius* L.).

**Fig. 3:**
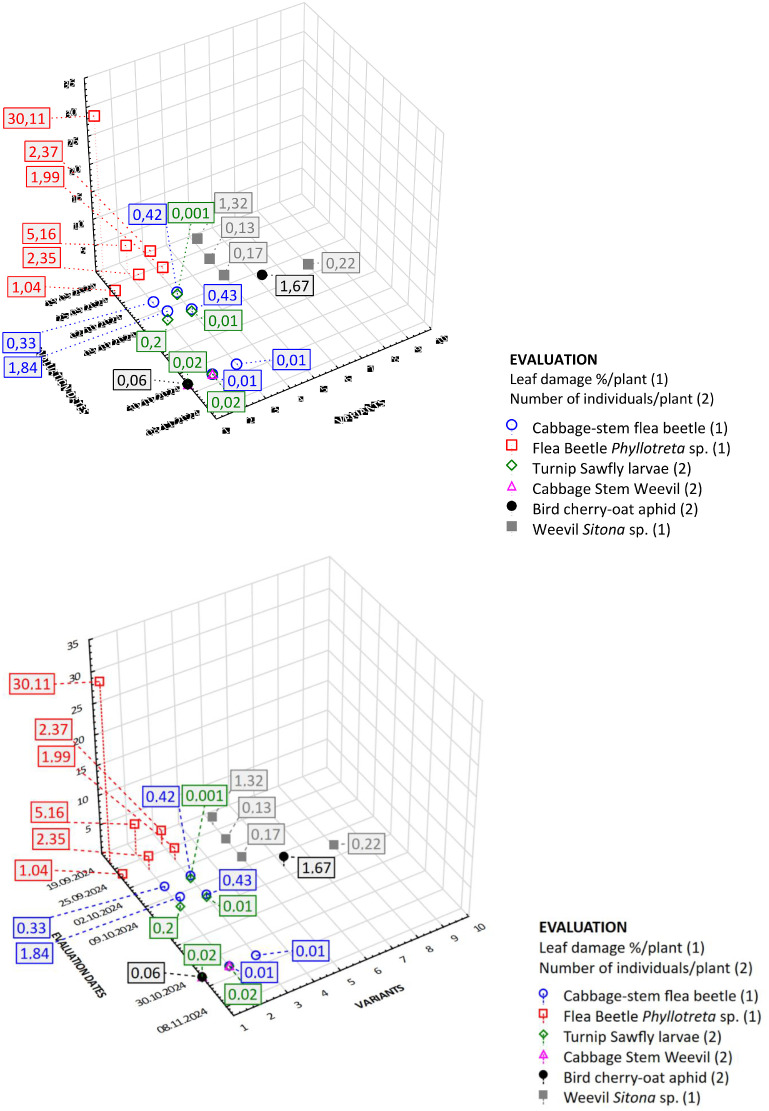
3D Scatterplot of multiple variables against evaluation dates and variants (cover crops) in 2024. Variants: 1 Watercress (*Lepidium sativum* L.), 2 Oil radish (*Raphanus sativus* L.), 3 Mustard (*Sinapis alba* L.), 4 Purple tansy (*Phacelia tanacetifolia* B.), 5 Crimson clover (*Trifolium incarnatum* L.), 6 Millet (*Panicum miliaceum* L.), 7 Buckwheat (*Fagopyrum esculentum* L.), 8 Fenugreek (*Trigonella foenum - graecum* L.), 9 Hungarian vetch (*Vicia pannonica Crantz*.), 10 Safflower (*Carthamus tinctorius* L.).

The Dataset brings information about the occurrence of six key insect pests and damage caused by them on ten cover crops species during three seasons in the period 2022–2024. Time series of data about the occurrence of pests and the corresponding degree of damage to leaf area or about the number of individuals converted to one plant are available for each cover crop species (Fig. 1, Fig. 2, Fig. 3).

Table 1, Table 2 and Table 3 present the average values of Crop Susceptibility Index (CSI) in ten cover crop species. Each variant brings:

• average CSI values calculated from the set **including zero occurrences and damage to plants** (*incl. zeros*) (Table 1) and from the set **including only non-zero values** (*non-zeros*) (Table 2),
• corresponding **95** **% confidence intervals** (Lower and Upper 95 % CI),
• **sequence** (Rank) by the average CSI value,
• **category of susceptibility** (Class) and
• **index of stability** (Δ) expressed as absolute difference between average CSI values calculated from the two sets (Table 3).

**Table 1:** Average Crop Susceptibility Index (CSI), 95 % confidence intervals and susceptibility class for zero values.

Rank (incl. zeros)	Variant	Average CSI (incl. zeros)	Lower 95 % CI (incl)	Upper 95 % CI (incl)	Class (incl. zeros)
1	Buckwheat	0	0	0	Very low
1	Hungarian vetch	0	0	0	Very low
1	Purple tansy	0	0	0	Very low
4	Millet	0.018510	0.001740	0.043406	Very low
5	Fenugreek	0.020833	0	0.052083	Very low
6	Safflower	0.023438	0	0.050781	Very low
7	Crimson clover	0.024779	0.007181	0.049402	Very low
8	Mustard	0.069024	0.033264	0.107255	Very low
9	Watercress	0.070151	0.013220	0.143750	Very low
10	Oil radish	0.081619	0.044473	0.121780	Very low

**Table 2:** Average Crop Susceptibility Index (CSI), 95 % confidence intervals and susceptibility class for non-zero values.

Rank (non-zeros)	Variant	Average CSI (non-zeros)	Lower 95 % CI (non)	Upper 95 % CI (non)	Class (non-zeros)
1	Buckwheat	0	0	0	Very low
1	Hungarian vetch	0	0	0	Very low
1	Purple tansy	0	0	0	Very low
5	Millet	0.074042	0.023083	0.131958	Very low
10	Fenugreek	0.166667	0.166667	0.166667	Very low
8	Safflower	0.125000	0.104167	0.166667	Very low
4	Crimson clover	0.049558	0.020202	0.087910	Very low
6	Mustard	0.092032	0.050092	0.134532	Very low
9	Watercress	0.160346	0.056407	0.298441	Very low
7	Oil radish	0.100454	0.060634	0.140641	Very low

**Table 3:** Stability ranking (Δ).

Variant	Delta (non - incl)	Stability class (Δ)
Buckwheat	0	Stable
Hungarian vetch	0	Stable
Purple tansy	0	Stable
Millet	0.055531	Stable
Fenugreek	0.145833	Moderate gap
Safflower	0.101563	Moderate gap
Crimson clover	0.024779	Stable
Mustard	0.023008	Stable
Watercress	0.090195	Stable
Oil radish	0.018835	Stable

There is also **stability class** (Stability class) determined by the value of index Δ (Table 3).

All values are presented as means calculated from the observations of individual cover crop variants.

Furthermore, the values represent the average susceptibility index (CSI) of plants ± 95 % confidence intervals (CI) calculated for ten cover crop species. The sequence (Rank) was determined separately for the data sets including zero occurrences and damage (“incl. zeros“) and for the data sets including only non-zero occurrences and damage (“non-zeros“). Index Δ (Table 3) expresses an absolute difference between the two average CSI values and serves to determine the stability class (Stable, Moderate gap). All variants were classified in the “Very low” class (very low susceptibility). The used data set included one aggregated value for each combination of respective variants (cover crop × year × evaluation date × pest). Neither the replications of plots nor the raw data on individual plants were available. For this reason, uncertainty was quantified using CSI by means of bootstrap resampling across the repeated evaluation data for the individual variants, i.e. cover crops (Table 4).

**Table 4:** Mean Composite Susceptibility Index (CSI) and percentile-based 95 % bootstrap confidence intervals (*n* = 10,000; seed = 12,345) for ten cover crop species (2022–2024).

Variant	Mean Include Zeros	2.50 %	97.50 %	Mean Non Zeros	2.50 %	97.50 %
Watercress	0.07002	0.01322	0.14375	0.16097	0.05641	0.29844
Oil radish	0.08191	0.04447	0.12178	0.10035	0.06063	0.14064
Mustard	0.06896	0.03326	0.10726	0.09160	0.05009	0.13453
Purple tansy	0,00,000	0.00000	0.00000	0.00000	0.00000	0.00000
Crimson clover	0.02497	0.00718	0.04940	0.04940	0.02020	0.08791
Millet	0.01871	0.00174	0.04341	0.07378	0.02308	0.13196
Buckwheat	0.00000	0.00000	0.00000	0.00000	0.00000	0.00000
Fenugreek	0.02063	0.00000	0.05208	0.16667	0.16667	0.16667
Hungarian vetch	0.00000	0.00000	0.00000	0.00000	0.00000	0.00000
Safflower	0.02351	0.00000	0.05078	0.12496	0.10417	0.16667

Comment: Mean CSI value was determined using non-parametric bootstrap (10,000 iterations, seed = 12,345); 95 % confidence intervals were determined using the percentile method (2.50 % and 97.5 %) from bootstrap distribution. This approach does not assume a symmetry of selective distribution and is therefore suitable exactly for data with the excessive occurrence of zero values and for data without normal distribution. The presented intervals express the variability of CSI index across the repeated evaluation dates.

## 4 Experimental Design, Materials and Methods

**Fig. 4:**
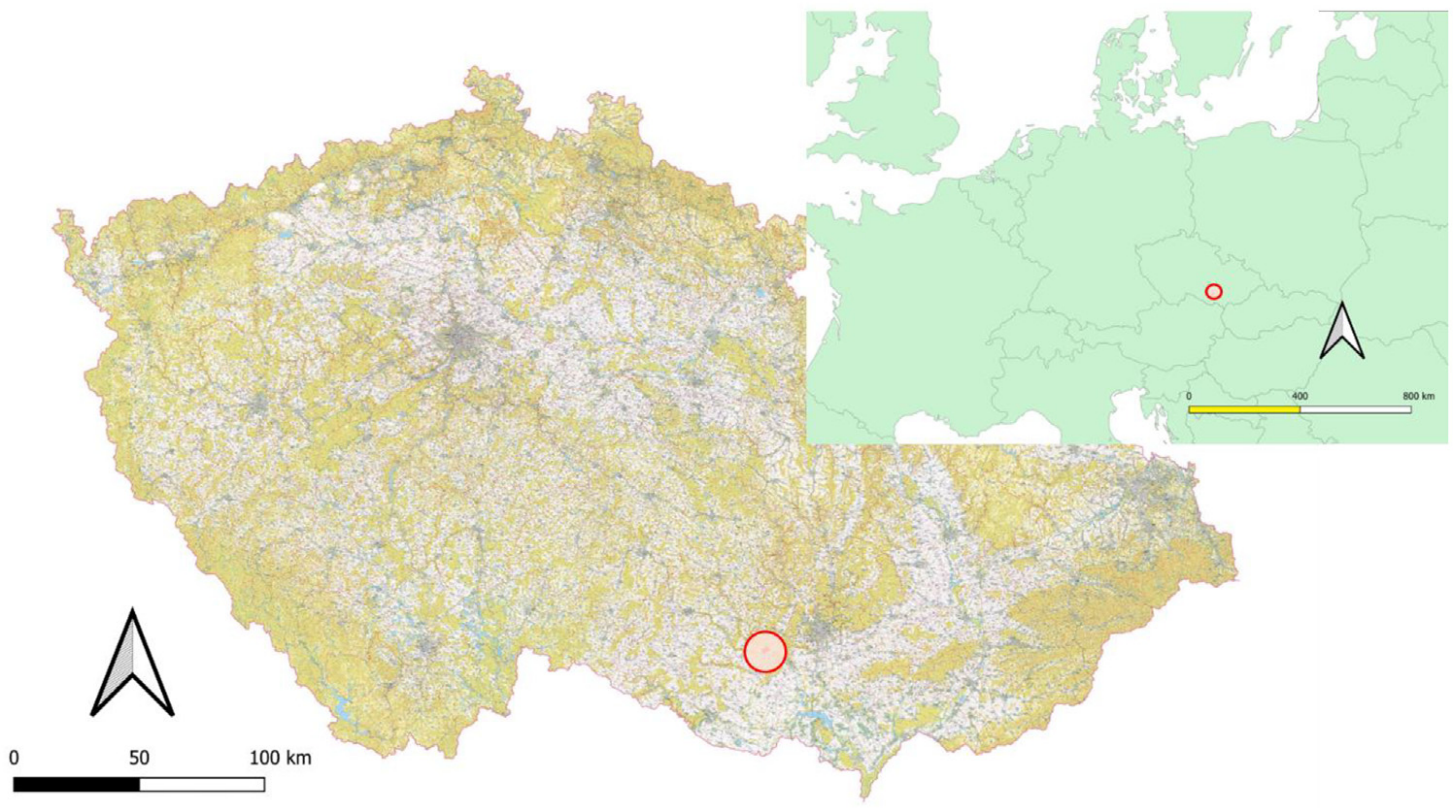
Map of field experiment sites in the Czech Republic (prepared based on Kintl et al. [2]).

In the period from September to November 2022–2024, the occurrence of six insect pest species (Table 5) was monitored on ten cover crop species grown as pure cultures in the area of interest (Fig. 4) – an overview and affiliation to the families of these cover crops are presented in Table 6. Details of experimental site are presented in Site characteristics.

**Table 5:** Overview of monitored insect pests and methods of their evaluation.

	Pest	Evaluation method
1	Cabbage-stem flea beetle (*Psylliodes chrysocephala* L.)	leaf damage % per plant
2	Flea Beetle *Phyllotreta* sp.	leaf damage % per plant
3	Turnip Sawfly larvae (*Athalia rosae* L.)	number of individuals per plant
4	Cabbage Stem Weevil (*Ceutorhynchus pallidactylus* Marsham)	number of individuals per plant
5	Bird cherry-oat aphid (*Rhopalosiphum padi* L.)	number of individuals per plant
6	Weevil *Sitona* sp.	leaf damage % per plant

**Table 6:** Overview of assessed variants.

No.	Variant	Family
1	Watercress (*Lepidium sativum* L.)	*Brassicaceae*
2	Oil radish (*Raphanus sativus* L.)	*Brassicaceae*
3	Mustard (*Sinapis alba* L.)	*Brassicaceae*
4	Purple tansy (*Phacelia tanacetifolia* B.)	*Boraginaceae*
5	Crimson clover (*Trifolium incarnatum* L.)	*Fabaceae*
6	Millet (*Panicum miliaceum* L.)	*Poaceae*
7	Buckwheat (*Fagopyrum esculentum* L.)	*Polygonaceae*
8	Fenugreek (*Trigonella foenum-graecum* L.)	*Fabaceae*
9	Hungarian vetch (*Vicia pannonica* Crantz.)	*Fabaceae*
10	Safflower (*Carthamus tinctorius* L.)	*Asteraceae*

These species were chosen with respect to the diversity of botanic families and agrotechnical significance in the conditions of changing climate. Watercress (*Lepidium sativum* L.), Oil radish (*Raphanus sativus* L.) and Mustard (*Sinapis alba* L.) belong in the family of *Brassicaceae*. The objective was to find out whether, to what extent and harmfulness insect pests occur in the given cover crops, which are of key importance for widely spread winter rape. Purple tansy (*Phacelia tanacetifolia* Benth.), Crimson clover (*Trifolium incarnatum* L.), Millet (*Panicum miliaceum* L.), Buckwheat (*Fagopyrum esculentum* L.), Fenugreek (*Trigonella foenum-graecum* L.), Hungarian vetch (*Vicia pannonica* Crantz.) and Safflower (*Carthamus tinctorius* L.) which are species not belonging in the *Brassicaceae* family and are therefore useful for crop rotation with rape or with other crops from this family. Two species (Crimson clover and Hungarian vetch) are legumes fixing atmospheric nitrogen. Other two species (Purple tansy and Buckwheat) belong in families that are not related to commonly grown crops (*Boraginaceae* and *Polygonaceae*), Safflower belongs in the family of *Asteraceae,* and the last species (Millet) belongs in the family of *Poaceae*.

### 4.1 Site characteristics

The research was conducted in 2022–2024 in the municipality of Nová Ves within a pilot testing of selected cover crop species (Fig. 4).

In terms of agro-ecological division, experimental plots are situated in the beet production region of the Czech Republic (CZ) which is a part of the European Union (EU), and their location is shown on the map (Fig. 4). Geographical coordinates of individual experimental fields were as follows:, 49.109583 N, 16.332938 E (2022; experimental field no 9701/16), 49.110080 N, 16.331470 E (2023; experimental field no 0901/8), 49.101287 N, 16.310569 E (2024; experimental field no 1801/8). Distance between the individual experimental plots was approximately 750 m. The plots did not exhibit any essential differences in soil and agrochemical properties. In all years, the total area of each experimental variant was 12 × 100 m. No plant protection preparations were used before sowing or during the experiment.

The Czech Research Institute for the Monitoring and Protection of Soils and Water Resources informed about the pedological characteristics of experimental plots to be as follows: brown earths (Cambisols) occurring mainly on flat terrains, with omnidirectional slope and total soil skeleton content of up to 10 %. Geological subsoil in this area is loess loam and loess clay of the Bohemian Massif, a smaller part of the area is formed of calcium clays with the occurrence of sands at some places, which belong in the Carpathian system. Soil type is Haplic Cambisol. As the field experiment was conducted in operating conditions of the farm, winter wheat could not be grown on the same plot in consecutive years. This is why the experiments were conducted on different plots in the same locality (Fig. 4) with identical soil and chemical conditions characterized above (Table 7). After the harvest of wheat, the stubble was processed to a depth of 8 cm and post-harvest residues were shallowly incorporated into the topsoil.

**Table 7:** Basic agrochemical parameters of experimental fields (prepared based on Kubíková et al. [1]).

Season	2022	2023	2024
**Soil reaction and plant available nutrient content**	**Experimental field no 9701/16**	**Experimental field no 0901/8**	**Experimental field no 1801/8**

pH (CaCl_2_)	6.4	6.7	7.4
P (mg kg^−1^)	232	256	259
Ca (mg kg^−1^)	3 620	4 067	7 993
K (mg kg^−1^)	381	379	302

Comment: The presented values originate from the national Agrochemical Soil Testing Programme of the Czech Republic, conducted by the Central Institute for Supervising and Testing in Agriculture. Plant-available nutrient contents were analysed using the Mehlich III extraction method, and soil exchangeable acidity (pH) was determined in a CaCl₂ extract. All laboratory procedures were carried out in compliance with the Czech Regulation No 275/1998, which defines the official standards for agrochemical soil analysis.

The field experiments were established in the pilot conditions of conventional farm within a standard crop rotation. In all years, the experiments with cover crops were conducted after the same pre-crop which was winter wheat (*Triticum aestivum* L.) to eliminate a possible influence of another pre-crop.

Sowing dates of cover crops were 10 August 2022, 21 August 2023 and 25 August 2024. The cover crops were sown using the Lemken Solitair 9 seeder. The growing period of cover crops was from August to the end of autumn or until the next year spring depending on the crop species (susceptibility to frost damage). In August 2023, the growing period of cover crops was characterized by lower temperatures and high precipitation. In August 2022 and 2024, the temperature was higher and precipitation lower.

**Fig. 5:**
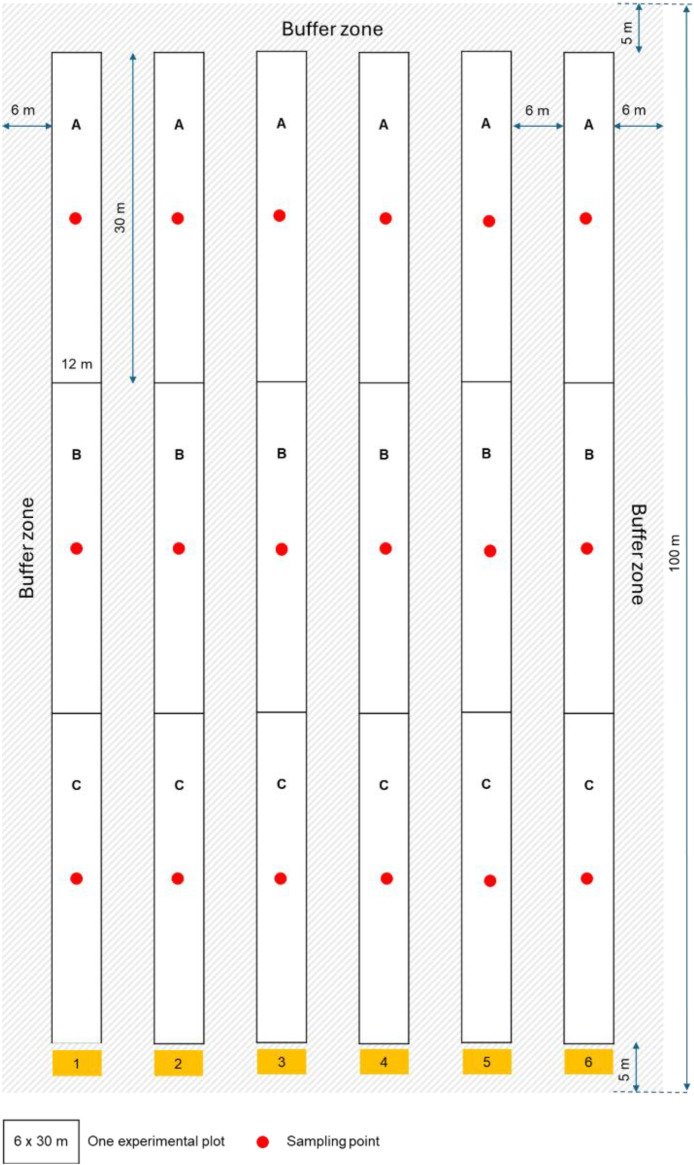
Schematic representation of the experimental layout, sampling points, and plant biomass collection method (prepared based on Kintl et al. [2]).

Individual experimental variants were arranged in three blocks, and fixed sampling points were established within each block using GPS coordinates to ensure consistent spatial reference throughout the study (Fig. 5). From these georeferenced points, plant biomass was collected following the methodology described in Kintl et al. [1], allowing for standardised and reproducible sampling across all experimental years. On the respective experimental plots (1–6), the following cover crops species were grown according to the predefined design, which remained identical for the entire duration of the field experiment (2022–2024, Table 5): 1 - Watercress (*Lepidium sativum* L.), 2 - Oil radish (*Raphanus sativus* L.), 3 - Mustard (*Sinapis alba* L.), 4 - Purple tansy (*Phacelia tanacetifolia* B.), 5 - Crimson clover (*Trifolium incarnatum* L.), 6 - Millet (*Panicum miliaceum* L.) etc.. This uniform scheme ensured comparable results throughout the years and enabled the assessment of interannual variability in crop performance. The consistent spatial layout and repeated cultivation of identical cover crop variants also allowed for a robust evaluation of treatment effects under varying weather and soil conditions.

**Fig. 6:**
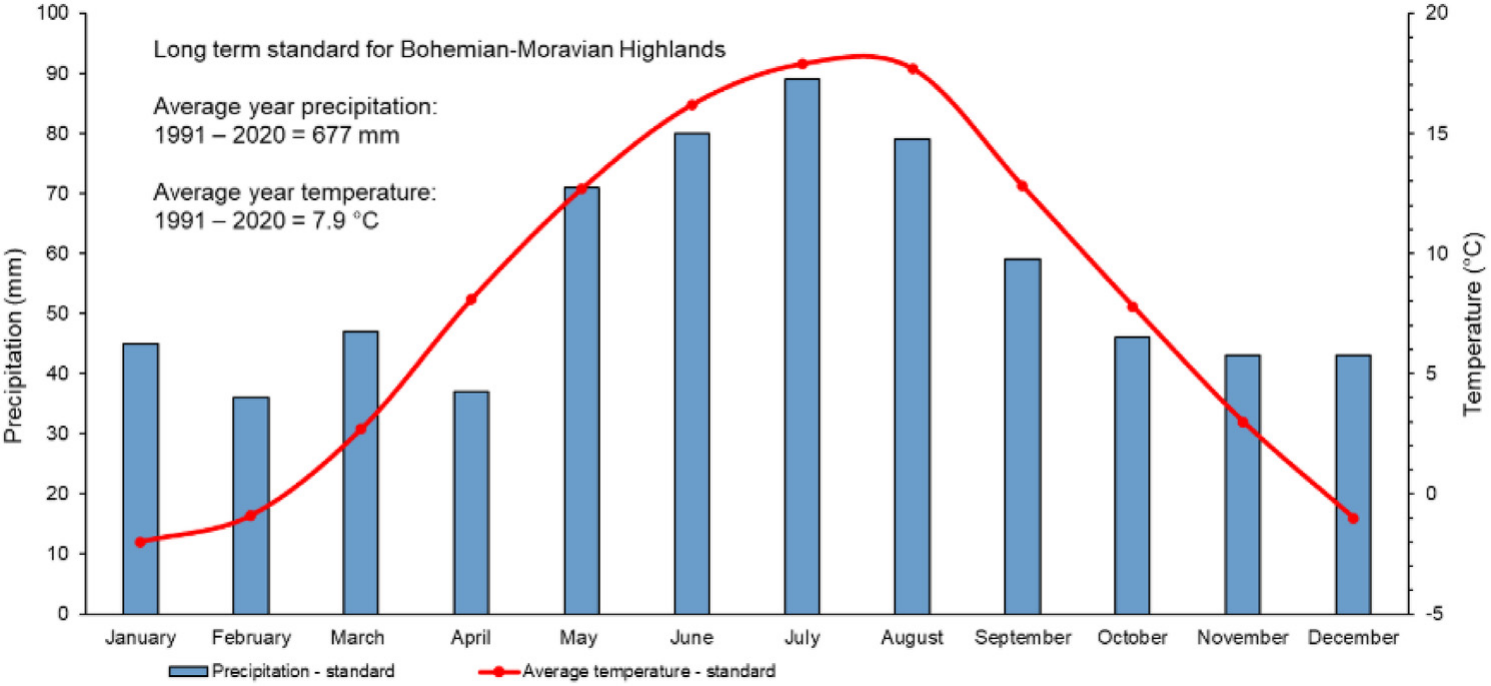
Climate diagram for the area of interest (prepared based on Kubíková et al. [1]).

Weather conditions during the 2021/2022 to 2024/2025 growing seasons are documented using basic meteorological parameters (Table 8) and a climate diagram (Fig. 6). The cover-crop growing periods in 2022 and 2023 were characterised by lower air temperatures and high precipitation in August, which generally supported early biomass accumulation due to sufficient soil moisture availability. In contrast, August 2022 and 2024 exhibited higher temperatures accompanied by reduced precipitation, resulting in temporary moisture deficits that likely constrained early growth and subsequently reduced biomass production.

**Table 8:** Basic meteorological parameters of research site for 2021–2024 (prepared based on Kubíková et al. [13]).

Indicator	Year/ month	1	2	3	4	5	6	7	8	9	10	11	12
Average monthly temperatures (°C)	2022	1.3	3.7	3.8	8.0	15.7	20.3	20.6	20.9	13.4	10.9	4.9	0.4
	2023	2.8	2.3	5.6	8.0	13.8	18.7	21.8	19.9	17.8	12.1	4.9	1.9
	2024	0.2	6.1	8.1	11.1	15.8	19.4	21.7	22.1	16.2	10.4	3.1	1.7
Maximum monthly temperatures (°C)	2022	12.8	11.6	19.2	21.1	28.7	32.6	34.2	33.2	25.3	22.2	16.3	10.0
	2023	11.4	13.9	20.1	19.7	25.1	31.5	32.8	33.1	29.1	25.0	15.4	12.1
	2024	11.4	14.1	20.6	25.6	24.9	33.0	34.0	34.2	33.2	19.5	13.1	10.4
Minimum monthly temperatures (°C)	2022	−7.8	−6.8	−9.1	−1.1	9.1	13.4	13.6	14.1	4.6	1.4	−2.5	−7.3
	2023	−6.0	−9.6	−7.0	−1.4	7.0	11.9	13.7	10.7	10.1	1.1	−5.4	−11.7
	2024	−12.3	−2.8	−3.4	1.8	10.9	11.0	14.2	14.4	5.1	2.1	−5.0	−5.5
Monthly precipitation total (mm)	2022	12.0	8.5	10.7	12.3	74.1	33.7	56.0	80.6	37.0	11.5	13.3	47.3
	2023	27.2	16.6	15.6	78.3	52.9	32.0	21.4	116.9	19.8	24.2	52.3	70.7
	2024	39.2	21.2	38.6	18.1	118.2	155.8	58.8	35.3	193.6	27.1	11.1	25.4

Comment: Table 11 provides mean air temperatures and total precipitation amounts recorded in each experimental year (2022–2024). Weather data were obtained from a professional meteorological station operated by the Czech Hydrometeorological Institute, equipped with the following instruments: rain gauge MR3H-FC (METEOSERVIS Ltd., Czech Republic), wind gauge WAA151 (VAISALA Ltd., Finland), and temperature and humidity sensor HMP35D (VAISALA Ltd., Finland).

### 4.2 Field evaluation

The evaluation dates are presented in Table 6. The records include the crop development stage (Table 9, Table 10, Table 11, Table 12) according to Biologische Bundesanstalt, Bundessortenamt, and CHemical industry, i.e. BBCH phenology scale according to Meier [3]. The visual determination of pests (Table 5) occurring in the stand was followed by the assessment of % damage to plant leaves in the respective variants (Table 6). The evaluation was made at 4 places in 50 plants, i.e. in total for the variant of 200 plants. The percentage of leaf area damage was converted to one plant and the number of individuals (caterpillars and adults) was converted to one plant too.

**Table 9:** Evaluation dates in the respective years.

No.	2022	2023	2024
1	30 Aug 2022	13 Sept 2023	19 Sept 2024
2	15 Sept 2022	20 Sept 2023	25 Sept 2024
3	30 Sept 2022	4 Oct 2023	2 Oct 2024
4	13 Oct 2022	26 Oct 2023	9 Oct 2024
5		2 Nov 2023	30 Oct 2024
6		21 Nov 2023	8 Nov 2024

**Table 10:** BBCH development stages of variants on evaluation dates in 2022.

No.	Variant	2022			
		30 Aug	15 Sept	30 Sept	13 Oct
1	Watercress (*Lepidium sativum* L.)	10–19	19–59	59–65	65
2	Oil radish (*Raphanus sativus* L.)	11–19	16–19	35	37
3	Mustard (*Sinapis alba* L.)	10–19	59	59–61	59–61
4	Purple tansy (*Phacelia tanacetifolia* B.)	14–16	19	39–59	32–33
5	Crimson clover (*Trifolium incarnatum* L.)	11–13	15–19	29	29
6	Millet (*Panicum miliaceum* L.)	10–16	16–59	17–59	19–59
7	Buckwheat (*Fagopyrum esculentum* L.)	10–19	59–61	65	65
8	Fenugreek (*Trigonella foenum - graecum* L.)	11–13	19–24	19–32	59
9	Hungarian vetch (*Vicia pannonica* Crantz.)	10–14	19	19–32	29–32
10	Safflower (*Carthamus tinctorius* L.)	10–14	14–32	19–35	35–59

**Table 11:** BBCH development stages of variants on evaluation dates in 2023.

No.	Variant	2023					
		13 Sept	20 Sept	4 Oct	26 Oct	2 Nov	21 Nov
1	Watercress (*Lepidium sativum* L.)	19	29–35	59–65	69–73	69–75	69–79
2	Oil radish (*Raphanus sativus* L.)	19	29	29–31	45	59	59–65
3	Mustard (*Sinapis alba* L.)	25	49–51	59–61	65–71	69–75	65–75
4	Purple tansy (*Phacelia tanacetifolia* B.)	19	29	35	59	59	59–65
5	Crimson clover (*Trifolium incarnatum* L.)	13	13–15	19	29	29	29
6	Millet (*Panicum miliaceum* L.)	14–29	19–55	55	75	80	85
7	Buckwheat (*Fagopyrum esculentum* L.)	19–51	61	65	75	85	85
8	Fenugreek (*Trigonella foenum - graecum* L.)	11–15	19–29	19–31	59	59–61	59
9	Hungarian vetch (*Vicia pannonica* Crantz.)	13–16	19–32	32	32	32	32
10	Safflower (*Carthamus tinctorius* L.)	14–16	15–29	39	39	45–55	59

**Table 12:** BBCH development stages of variants on evaluation dates in 2024.

No.	Variant	2024					
		19 Sept	25 Sept	2 Oct	9 Oct	30 Oct	8 Nov
1	Watercress (*Lepidium sativum* L.)	10–14	12–32	12–34	16–59	16–65	16–65
2	Oil radish (*Raphanus sativus* L.)	10–16	12–16	13–29	14–29	29	32
3	Mustard (*Sinapis alba* L.)	10–16	14–32	12–39	12–53	59	59
4	Purple tansy (*Phacelia tanacetifolia* B.)	12–16	12–19	12–19	14–29	39	39
5	Crimson clover (*Trifolium incarnatum* L.)	10–14	11–15	12–19	12–19	29	29
6	Millet (*Panicum miliaceum* L.)	10–13	13–29	12–33	13–33	55–59	55–59
7	Buckwheat (*Fagopyrum esculentum* L.)	12–51	12–61	59–65	65	73	73
8	Fenugreek (*Trigonella foenum - graecum* L.)	10–14	12–19	12–19	12–16	32	32
9	Hungarian vetch (*Vicia pannonica* Crantz.)	10–14	10–15	10–17	10–29	29	29
10	Safflower (*Carthamus tinctorius* L.)	12–13	12–16	12–17	14–16	29–33	29–33

### 4.3 Statistical data processing

The data were processed in the Statistica 14 programme (TIBCO Software, Inc., Palo Alto, SF, USA) and in Microsoft Excel.

Prior to being analysed, all variables **were standardized using the min-max method (0–1)** with the lowest value of each variable being converted to 0 and the highest value being converted to 1 [4]. This procedure made it possible to eliminate the effect of different units (number of individuals vs. percentage of damage) betwween the monitored pests and to allow their mutual comparison.

$$x^{\prime }=\left(x-x_{min}\right)/\left(x_{max}-x_{min}\right)$$

where

*x’* is standardized value,

*x* original value,

*x_min_* and *x_max_* minimum and maximum values detected in the given pest.

The result is a dimensionless value in the 0–1 interval, which allows biological interpretation (0 = pest absence, 1 = maximum infestation).

Then the **Composite Susceptibility Index CSI** was calculated for each date of valuation as the arithmetic mean of six standardized values. This procedure corresponds to general principles of creating multimetric (composite) ecological indices used for the complex expression of ecological response to multiple factors at the same time [[5], [6], [7]]. Data preparation and the selection of individual categories were made according to Kim et al. [8]. The calculation was then made according to Kim et al. [8] and Murthy et al. [9].

$$CSI_{j}=1/n\sum_{i=1}^{n}x\prime_{ij}$$

where

*n* = number of monitored pests (6), x´_ij_ = standardized value for pests *i* in the cover crop *j*.

Two statistical approaches were applied:

(1) Calculation comprising all values including the zero ones representing stands not damaged by pests (**incl. zeros**). The values express a **total risk** of infestation and damage in the monitored period.
(2) Calculation from **non-zeros** only, i.e. from the damaged plants. The values represent **damage intensity** when a pest occurs.

**Non-parametric bootstrap** with 10 000 iterations and fixed seed (*seed* = 12,345) at the level of mean CSI index was used to express the statistical uncertainty and variability between the cover crop species. Based on the bootstrap division, **95** **% confidence intervals (CI)** were determined [10]. This approach makes it possible to estimate the variance and confidence interval even in case that the data does not meet the assumptions of parametric methods.

The values of Composite Susceptibility Index CSI were interpreted by categories in the extent from 0 to 1 using a four-point scale corresponding to the principle of *equal interval classification* used in composite indicators [11,12]:

• 0.00–0.20 = Very low
• 0.21–0.40 = Low
• 0.41–0.60 = Medium,
• 0.61–1.00 = High

Additionally, **a stability index Δ** was introduced, defined as an absolute difference between the mean CSI non-zero values (*non-zeros*) and CSI zero values *(incl. zeros)*. This indicator expresses the degree of difference between the potential and the actually observed susceptibility and makes it possible to distinguish the actually stable species from those with the low CSI index which follows out primarily from a high share of zero values detected in the observations.

Limit stability values were determined analogically to thresholds used in the evaluation of ecological stability and resilience.

• Stable (Δ ≤ 0.10),
• Moderate gap (0.11–0.20),
• High gap (0.21–0.40),
• Extreme gap (> 0.40).

The procedure allows to distinguish the actually resilient species from those with the seeming stability which follows out mainly from the high share of observed zero values.

Sowing dates of cover crops were 10 August 2022, 21 August 2023 and 25 August 2024. The cover crops were sown using the Lemken Solitair 9 seeder. The growing period of cover crops was from August to the end of autumn or until spring next year depending on the crop species (susceptibility to frost damage). In August 2023, the growing period of cover crops was characterized by lower temperatures and high precipitation. In August 2022 and 2024, the temperature was higher and precipitation lower.

## Limitations

Not applicable.

## Ethics Statement

The authors have read and follow the *ethical requirements* for publication in Data in Brief and confirm that the current work does not involve human subjects, animal experiments, or any data collected from the social media platforms.

## CRediT Author Statement

**Julie Sobotková:** Investigation. **Pavel Kolařík:** Methodology, Validation, Writing - Original Draft. **Antonín Kintl:** Conceptualization, Data Curation, Funding acquisition, Supervision. **Ondřej Malíček:** Investigation. **Oldřich Látal:** Investigation. **Igor Huňady:** Formal analysis, Methodology, Visualization. **Zuzana Kubíková:** Investigation, Writing - Review & Editing. **Martina Dočkalíková:** Conceptualization, Supervision. **Jakub Elbl:** Conceptualization, Data Curation, Visualization, Writing - Review & Editing.

## Data Availability

Mendeley DataData for the monitoring the occurrence of pests on catch crops (Original data).
